# Real-time PCR detection of the *HhaI* tandem DNA repeat in pre- and post-patent *Brugia malayi* infections: a study in Indonesian transmigrants

**DOI:** 10.1186/1756-3305-7-146

**Published:** 2014-03-31

**Authors:** Anna Albers, Erliyani Sartono, Sitti Wahyuni, Maria Yazdanbakhsh, Rick M Maizels, Ute Klarmann-Schulz, Kenneth Pfarr, Achim Hoerauf

**Affiliations:** 1 Institute of Medical Microbiology, Immunology and Parasitology, University Hospital of Bonn, Sigmund-Freud-Str. 25, D-53105 Bonn, Germany; 2 Department of Parasitology, Leiden University Medical Center, P.O Box 9600, 2300, RC Leiden, The Netherlands; 3 Parasitology Department, Medical Faculty, Hasanuddin University, Jl. Perintis Kemerdekaan 10 Tamalanrea, Makassar 90245, Indonesia; 4 Institute for Immunology & Infection Research, University of Edinburgh, Ashworth Laboratories, Edinburgh EH9 3JT, UK; 5 Institute for Medical Biometry, Informatics, and Epidemiology, University Hospital of Bonn, Sigmund-Freud-Str. 25, D-53105 Bonn, Germany

**Keywords:** *Brugia malayi*, amicrofilaremic, real-time PCR, *HhaI*, pre-patent, detection

## Abstract

**Background:**

Lymphatic filariasis caused by *Wuchereria bancrofti* or *Brugia* spp. is a public health problem in developing countries. To monitor bancroftian filariasis infections, Circulating Filarial Antigen (CFA) test is commonly used, but for brugian infections only microfilariae (Mf) microscopy and indirect IgG4 antibody analyses are available. Improved diagnostics for detecting latent infections are required.

**Methods:**

An optimized real-time PCR targeting the brugian *HhaI* repeat was validated with plasma from microfilariae negative Mongolian gerbils (jirds) infected with *B. malayi*. Plasma samples from microfilaremic patients infected with *B. malayi* or *W. bancrofti* were used as positive and negative controls, respectively. PCR results of plasma samples from a transmigrant population in a *B. malayi* endemic area were compared to those of life-long residents in the same endemic area; and to IgG4 serology results from the same population. To discriminate between active infections and larval exposure a threshold was determined by correlation and Receiver-Operating Characteristics (ROC) curve analyses.

**Results:**

The PCR detected *HhaI* in pre-patent (56 dpi) *B. malayi* infected jirds and *B. malayi* Mf-positive patients from Central Sulawesi, Indonesia. *HhaI* was also detected in 9/9 elephantiasis patients. In South Sulawesi 87.4% of the transmigrants and life-long residents (94% Mf-negative) were *HhaI* PCR positive. Based on ROC-curve analysis a threshold for active infections was set to >53 *HhaI* copies/μl (AUC: 0.854).

**Conclusions:**

The results demonstrate that the *HhaI* PCR detects brugian infections with greater sensitivity than the IgG4 test, most notably in Mf-negative patients (*i.e*. pre-patent or latent infections).

## Background

Lymphatic filariasis (LF) affects approximately 120 million people in 73 endemic countries [1]. *Wuchereria bancrofti* accounts for ~90% of LF cases, while *Brugia malayi* and *B. timori* infections account for the remainder [2]. Approximately 15 million people with LF live in Southeast Asia [3]. Indonesia is part of the Global Programme to Eliminate Lymphatic Filariasis (GPELF), a multi-national initiative that aims to eliminate disease transmission by 2020. GPELF employs mass drug administration (MDA) to reduce filarial infection rates below those required for sustained transmission with the goal of permanently eliminating LF caused by *B. malayi* and *W. bancrofti*[4,5]. GPELF relies on diagnostic tests to map LF-endemic areas and to monitor the impact of MDA. Thick blood smear examination is used for routine diagnosis and prevalence studies in *Brugia* endemic countries [6,7]. This method detects microfilariae (Mf) in the peripheral blood but, due to the nocturnal periodicity of microfilaremia in these areas, requires night-time collection, which is unpopular with the local population. Furthermore, this method is relatively insensitive [8].

In bancroftian filariasis, circulating filarial antigen (CFA) can be measured in plasma by a rapid card test [9,10] or enzyme-linked immunosorbent assay (ELISA). CFA determination revealed that approximately twice as many individuals are CFA-positive (CFA+) than are microfilaremic, thus a proportion of Mf-negative people equal to the Mf-positive people harbors cryptic infections [11]. Visualizing adult worms by ultrasonography (USG) is also possible in *W. bancrofti* infections which is helpful for monitoring adult worm viability, e.g. after macrofilaricidal treatment [6].

However, for brugian LF, a specific antigen test like CFA does not exist and USG is unable to detect cryptic infections [12]. Exposure to brugian parasites is determined by brugian specific IgG4 serology using recombinant antigens in ELISAs [13,14] or rapid-format assays (BmR1-Brugia Rapid^™^[15] and CELISA [16]) with up to 95% sensitivity in Mf-positive samples. The ELISA format using soluble worm antigen (SWA) was shown to be more sensitive compared to the rapid-format which could be due to the mixture of antigens used in the ELISA. However, IgG4 serology has limited use in cross-sectional surveys since it does not differentiate latent or occult infections from exposure to infective larvae. With less sensitivity it is also cross-reactive with *W. bancrofti* related antibodies and therefore has limited use in co-endemic areas.

Real-time PCR is a powerful tool for high-throughput detection of parasite DNA and is increasingly replacing conventional-PCR and microscopy for diagnosis of several parasitic infections [17,18]. Using a real-time PCR assay targeting the *B. malayi HhaI* repeat, a sensitive and specific detection of nocturnally periodic *B. malayi* DNA in day blood samples was possible including 33% of Mf-negative samples in a highly endemic area [19]. Recently a multiplex real-time PCR assay for the *B. malayi HhaI* and *W. bancrofti* Long DNA Repeat was established for use in co-endemic areas [20]. This once more showed that real-time PCR assays provide a significant cost and labor saving procedure for disease monitoring efforts in endemic locations as part of the Global Progamme for the Elimination of Lymphatic Filariasis (GPELF) [20].

We evaluated the usefulness of the *B. malayi HhaI* PCR as a diagnostic test, especially focusing on pre-patent, latent (Mf-negative) and occult infections (seen after continued use of antifilarial drugs, i.e. post-patent), analyzing an Indonesian transmigrant population, previously analyzed for *B. malayi* infection using both IgG4 based tests [21,22]. Most studies on the dynamics of filarial infection have been carried out in populations that are life-long residents of endemic areas. In such situations, the history of exposure to parasites is hard to determine and correlate with different diagnostic approaches. This is only possible when a naive population becomes exposed to filarial infection abruptly. In Indonesia, a governmental transmigration program in the 1990’s relocated groups of people from the overcrowded islands of Java and Bali to under-populated areas on remote islands, which are often endemic for a multiplicity of infections. This transmigration policy provided a unique situation to study the development of infection in individuals with a known and equal length of exposure to infections. We present new results concerning the dynamics of brugian infection in a population with a known time of residence in an endemic area using a modified qPCR for *B. malayi HhaI* that was validated using a novel cohort of Mf-negative samples.

## Methods

### Ethics statement

Human participants: This study used archived material from a previous project that was reviewed and approved by the ethics committee of the Medical Faculty Hasanuddin University, Makassar, South Sulawesi, Indonesia [22]. In co-operation with the medical doctors and health workers of the local District Health Centre and the head of each transmigration unit or village, all residents were informed about the study and invited to participate. Written informed consent was obtained from all study participants or parents of children and juveniles before parasitological studies and blood taking in accordance with the guidelines of the Indonesian Department of Health and Human Services.

Plasma from uninfected European controls or *Toxoplasma* spp. and *Dirofilaria repens* infected patients was obtained from blood bank volunteers after informed consent was given. Use of the plasma for diagnosis by PCR and other methods was approved by the Ethics Committee of the University Hospital of Bonn.

The use of plasma from Ghanaian volunteers infected with other parasites (*W. bancrofti*, *Schistosoma* spp. and *Strongyloides stercoralis*) was approved by the Committee on Human Research Publication and Ethics at the University of Science and Technology in Kumasi, Ghana and by the Ethics Committee of the University Hospital of Bonn.

Use of animals: All animal protocols were approved by the University of Edinburgh Ethical Review Committee (Certificate of Designation PCD 60/2605) and submitted to the UK Home Office which issued Project Licence PPL60/3453 for animal studies in the investigator's laboratory. UK Home Office guidelines to minimize animal suffering were adhered to at all times.

### Participants

Plasma from *B. malayi* infected patients from Indonesia were from a larger cross-sectional study between 1990–1996 conducted in Budong-budong [21,22], a district of Mamuju Regency in South Sulawesi, Indonesia, endemic for periodic nocturnal *B. malayi*[23]. A transmigrant population had travelled to their new homesteads in groups coming from the same village or region in Bali or certain Lesser Sunda islands as part of the government-sponsored relocation program. Transmigrants from areas where filariasis is endemic (South Sulawesi and other Lesser Sunda islands) were excluded from the analysis. A total number of 247 transmigrants and 133 life-long residents (LLR) were enrolled in the study. For PCR analysis, 334 plasma samples were available for DNA extraction (Additional file 1).

### Detection *of B. malayi* infection

Venous blood samples (10 ml) were collected between 20:00–24:00 as described [22]. EDTA was added to the tubes to a final concentration of 0.05 M. After centrifugation, collected plasma was stored at -20°C until shipment to Europe and then stored at -70°C until use. Parasitological examination and detection of filarial specific IgG4 antibodies using BmR1 dipstick assay (Brugia Rapid™) were done in 2004 as previously described [21,22].

### DNA extraction and PCR

DNA was extracted from 100 μl plasma with a QIAcube® (Qiagen, Hilden, Germany) using the QIAamp® DNA Blood Mini Kit according to the blood and body fluid protocol. DNA was eluted in 100 μl AE-Buffer.

The PCR assay used a QuantiTect® custom assay with primers and minor groove binding probe (QuantiProbe®, Qiagen) to detect 120 bp of the *Brugia HhaI* repeat (Additional file 2) [19]. Target sequence copy numbers were calculated from the C_t_ values using an external standard curve generated with an *HhaI* plasmid dilution series that had 100% detection in samples with >200 copies/μl and 80% in samples with 20 copies/μl.

Samples containing very low amounts of DNA and negative for *HhaI* were measured in a second PCR assay using the QuantiTect® Virus NR Kit. The modified master mix allows the sample DNA to make up 50% of the PCR reaction and resulted in an improved detection limit of 2 *HhaI* copies/μl (Additional file 2). A test for inhibitors in the DNA samples was done with a reference plasmid of murine *IFN*-γ (Additional file 2). An internal PCR control detected the human β-*actin* gene (Additional file 2).

### DNA extraction from plasma of *B. malayi* infected jirds

Male adult jirds (*Meriones unguiculatus*) were bred and housed according to Home Office guidelines, United Kingdom. Jirds with a patent *B. malayi* infection were used as a source of venous blood during the pre-patent infection. Blood was collected by tail bleed from jirds on day 56 post infection with *B. malayi*. DNA was extracted from 100 μl of plasma using the QIAamp® DNA Mini Kit, eluted in 50 μl and quantified with the QuantiTect® Virus NR Kit using 2 μl or 10 μl sample DNA per reaction.

### DNA extraction from plasma used as negative controls

The specificity of the *HhaI* PCR assay was tested using plasma samples collected from volunteers after informed consent and extracted as above from the following: 10 *W. bancrofti* patients, 15 patients with other parasitic infections (*Toxocara* spp., *Schistosoma* spp., *Strongyloides stercoralis*, *Dirofilaria repens* or *W. bancrofti*) and 10 European blood donors without history of parasitic infection.

### Determination of threshold of active infections by real-time PCR

A threshold limit of *HhaI* copy numbers was determined to differentiate most-likely “active” infections (i.e. infections with live adult worms) from simple exposure to infective larvae. For this the Mf loads of Mf-positive (Central Sulawesi and South Sulawesi transmigrants, n = 49) and LE patients (Central Sulawesi, n = 9) were correlated to *HhaI* copies/μl and the Y-intercept was calculated by linear regression. To verify the results, further analysis was done by a Receiver Operating Characteristics (ROC) curve analysis using a binormal mixture model [24,25]. Individual samples were scored positive with active infection (Mf-positive, LE n = 58) or negative with possible exposure to infective or fourth-stage larvae (L3-L4) (n = 50, transmigrant samples from the ≤1 month and 2–4 months group, since L3 to adult development requires ~8 months). The Area Under the Curve (AUC) for the ROC-curve was calculated to measure the discriminatory power of the PCR assay [26]. A table of possible thresholds with true- and false-positive rates (%) was calculated from the coordinates of the ROC curve.

### Statistical methods

PASW 18.0 (IBM®, Somers NY, USA) was used to test the comparison of two binary variables with the Fisher’s exact test, to correlate two continuous parameters using Spearman’s rank test and to determine a threshold copy number by ROC-curve analysis. The ROC-curve procedure is a useful tool to evaluate the performance of classification schemes that categorize samples into one of two groups (i.e. *HhaI* copy numbers of Mf positive (infected) vs. *HhaI* copy numbers of Mf negative (exposed but uninfected)). The further the curve lies above the reference line, the more accurate the test (AUC). The table with curve coordinates reports the sensitivity (true positive rate) and the 1-specificity (false positive rate) for every possible cutoff. A cutoff of 0 is equivalent to assuming that everyone is positive, the highest cutoff assumes that everyone is negative. Both extremes are unlikely, the balance of sensitivity and 1-specificity is needed to select a cutoff.

The Cochran-Armitage test for trend was calculated with SAS 9.2 (SAS, Cary, NC, USA) to compare the trend of *HhaI* copy numbers between the 1–3 year and the 4–6 year residents.

## Results

To establish a method for detecting cryptic (pre-patent or latent) *B. malayi* infections, real-time PCR for *HhaI* was used to measure parasite DNA in plasma samples. Using 2 μl of a *HhaI* plasmid DNA dilution series and the Quantitect® Probe master mix, 100% (5/5) of the samples with 200 copies/μl were detected (C_t_ 30.5-31). The reaction detected as few as 2 copies/μl in 50% (5/10) of the samples (C_t_ 36.5-37). A detection rate of 80% (4/5) was achieved for 20 copies/μl and set as the PCR detection limit (C_t_ 34.5-35). The reaction efficiency was ≥ 85% (Additional file 3A). Negative controls did not result in a signal.

Samples with low DNA concentrations could lead to false negatives when only using 2 μl of sample DNA. To increase the sample volume to a maximum of 10 μl, a second PCR using the QuantiTect® Virus NR Kit was developed. Using 10 μl of the 2 copies/μl plasmid dilution, the reaction efficiency was 100% and detected 100% of the samples (5/5; C_t_ 33–34; Additional file 3B).

To validate the *HhaI* PCR assay used for detection of *B. malayi* infection in plasma, the real-time QuantiTect® PCR assay was used on plasma taken eight weeks after infection from nine infected and nine naive jirds. No Mf could be found at this time confirming a pre-patent status. Patent *B. malayi* infections in jirds can usually be detected 75–110 days after infection [27]. All infected jirds were confirmed to have adult worms (median = 40, range = 9–76) when the animals were necropsied. The *B. malayi HhaI* PCR assay using 10 μl reaction volume showed a positive signal in all infected jirds 8 weeks post infection, but was negative in plasma samples from uninfected jirds (Table 1). Using only 2 μl of template DNA with the QuantiTect master mix, there was no detection of the *HhaI* tandem DNA repeat. The results confirmed the sensitivity of the *HhaI* PCR when using an increased volume of template DNA in pre-patent, Mf-negative samples.

**Table 1:** ***HhaI *** **detection in plasma from pre-patent jirds (56 days post infection) 2 μl and 10 μl sample DNA volume**

**Animal group**	***HhaI *** **positives (2 μl sample volume)**	***HhaI *** **positives (10 μl sample volume)**	**Adult worm recovery**^**a**^
Infected jirds (9/9 patent)	0/9	9/9	40 (9–76)
Uninfected jirds	0/9	0/9	0

^a^Median (range).

Plasma samples of European blood donors, 19 samples with different parasite infections and 10 plasma samples from *W. bancrofti* infected patients were tested with the *HhaI* PCR 2 and 10 μl assays. These controls were all negative, confirming the specificity of the *HhaI* PCR primers and minor groove binding hybridization probe as previously reported [19].

The PCR was further validated using samples from 30 Mf-positive individuals from Central Sulawesi and 9 elephantiasis patients, one of whom was Mf-positive. The *HhaI* gene was detected in all Mf-positive samples confirming the sensitivity of the PCR in microfilaremic samples (positive controls). Copy numbers of the *HhaI* gene were positively correlated to the number of Mf (Spearman’s rank correlation, r = 0.48, *P* = 0.0072). In the elephantiasis group, the 2 μl PCR assay detected *HhaI* in 78% of the samples, although only one patient was Mf-positive with 1 Mf/ml (Table 2). In the 10 μl assay, all elephantiasis patient samples were positive for *HhaI* with a median of 57 copies/μl (range: 5–538; Additional file 4A) compared to Mf-positive samples with a median of 448 copies/μl (range 25–8252; Additional file 4B). The IgG4 test in LE patients was positive in only 3/9 samples (27%). Even though LE patients often lack Mf and are negative for CFA in *W. bancrofti* infections, it is assumed that LE patients in endemic areas have a constant exposure to incoming larvae (L3), which more strongly stimulate the hosts’ immune system, resulting in L3 killing (and thus no infection) at the expense of tissue damage. DNA from these L3 can be detected by the sensitive *HhaI* PCR.

**Table 2:** **Validation of the *HhaI *** **real-time PCR using positive control patients from Central Sulawesi, Indonesia**

**Infection status**	**PCR positive (2 μl sample volume)**	**%**	**PCR positive (10 μl sample volume)**	**%**	**IgG4 dipstick positive**	**%**
Mf positive patients (n = 30)	30/30	100	30/30	100	30/30	100
Elephantiasis patients						
(Mf-positive n = 1, Mf-negative n = 8)	7/9	78	9/9	100	3/9	27

After validating the sensitivity and specificity of the assay, plasma samples from 229 transmigrants in South Sulawesi and 105 life-long residents (LLR) were measured with the *HhaI* PCR. All 19 Mf-positive patients were positive for *HhaI* and IgG4 dipstick (Additional file 1).

**Figure 1:**
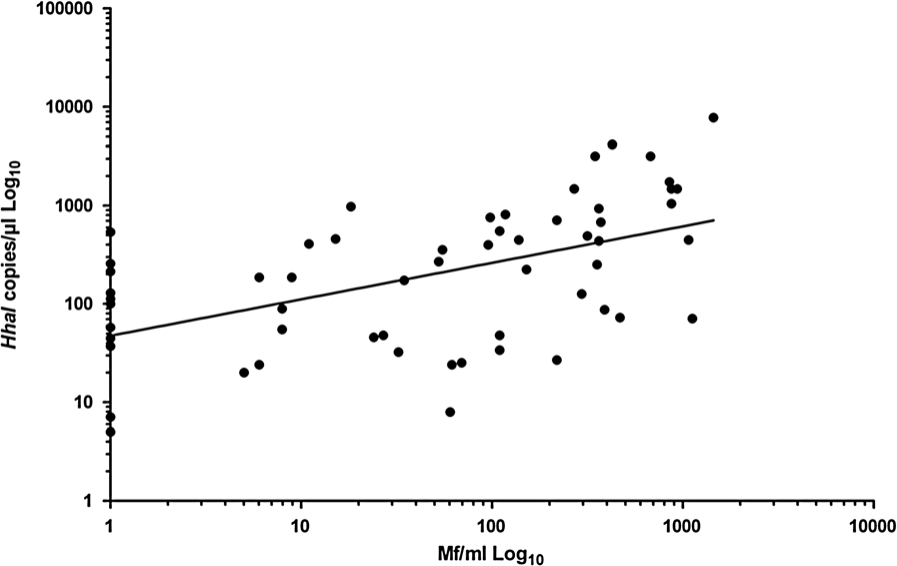
**Determination of a threshold for infection.** Mf loads from Mf-positive and LE patients were correlated to *HhaI* copies/μl (n = 58). Linear regression was performed to calculate the Y-intercept and standard deviation of the line. The threshold was defined as: Y-intercept (47.31) + 4XSD (1.43). Spearman Rank correlation resulted in r = 0.52 with *P* < 0.001 (PASW 18.0 software package).

Defining Mf-positive (Central Sulawesi and transmigrants) and LE samples as having or having had an active infection, the Mf numbers significantly correlated with *HhaI* copies/μl (Figure 1, Spearman rank correlation, r = 0.52, P < 0.001). The linear regression line from the correlation crossed the Y-axis at 47.31 *HhaI* copies/μl (SD = ±1.43) (Figure 1). The threshold of active infections was calculated to be 53.03 *HhaI* copies/μl (Y-intercept plus 4XSD) [28]. Transmigrant samples with *HhaI* copy numbers below the threshold were assumed to have DNA from L3 that were killed, but no active infection.

**Figure 2:**
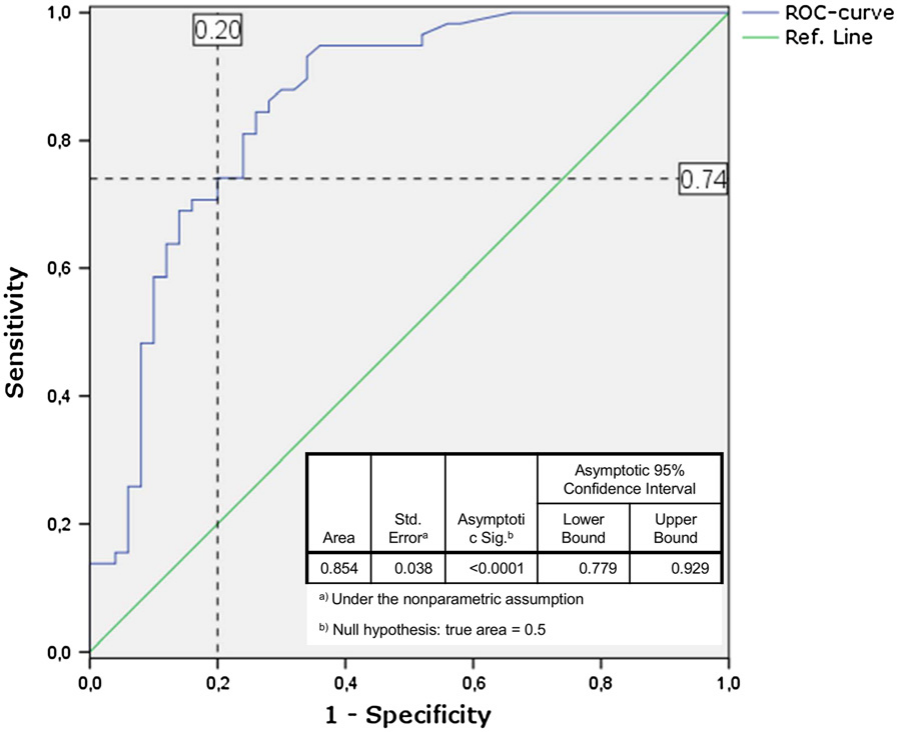
**ROC curve.***HhaI* copy numbers from infected individuals (n = 58) and non-infected individuals (n = 50) were plotted to create a ROC curve with corresponding coordinates of the curve and statistics for the Area Under the Curve (blue line). A possible threshold is shown in dashed lines with values (%) for Sensitivity (true positive rate) and 1-Specificity (false positive rate) (PASW 18.0 software package).

Using a ROC curve we were able to calculate possible thresholds with the coordinates of the curve (Figure 2). The variable *HhaI* copies/μl has at least one tie (at 53.5 *HhaI* copies/μl) between the sensitivity (true positive rate, Mf positive and infected) and the 1-specificity (false positive group, Mf-negative, uninfected/exposed). Therefore a threshold (cutoff) of 53.5 *HhaI* copies/μl was found to best discriminate between real infections and uninfected/exposed individuals based on PCR results.

ROC-curve analysis confirmed that the threshold determined with the correlation and regression line had good discriminatory power (AUC = 0.854; P < 0.001) (Figure 2). Using a cutoff of 53 copies/μl, 74.1% true-positive and 20% false-positive rates were calculated by ROC-curve analysis (Figure 2, Table 3). Based on these analyses, we set 53 *HhaI* copies as the cutoff to differentiate patients with an active infection (persons infected with adult worms) from those who only had been exposed to infective larvae.

**Table 3:** Coordinates of ROC curve

**Positive if greater than or equal To**	**Sensitivity (True positive)**	**1-Specificity (False positive)**
1.00	1.000	1.000
1.50	1.000	.760
2.50	1.000	.740
3.50	1.000	.720
4.50	1.000	.660
5.50	.983	.580
6.50	.983	.560
7.50	.966	.520
8.50	.948	.520
9.50	.948	.500
12.00	.948	.480
14.50	.948	.460
15.50	.948	.440
16.50	.948	.420
17.50	.948	.400
18.50	.948	.380
19.50	.948	.360
22.00	.931	.340
24.50	.897	.340
25.50	.879	.320
26.50	.879	.300
29.50	.862	.280
32.50	.845	.280
33.50	.845	.260
35.50	.828	.260
39.50	.810	.260
43.50	.810	.240
45.50	.793	.240
47.00	.776	.240
50.00	.741	.240
**53.50**	**.741**	**.200**
56.00	.724	.200
58.50	.707	.200
61.00	.707	.180
66.00	.707	.160
70.50	.690	.160
72.00	.690	.140
80.00	.672	.140
88.00	.655	.140
90.00	.638	.140
95.50	.638	.120
106.00	.621	.120
119.00	.603	.120
126.50	.586	.120
128.00	.586	.100
150.50	.569	.100
179.50	.552	.100
200.50	.517	.100
220.00	.500	.100
226.50	.483	.100
239.00	.483	.080
255.00	.466	.080
264.50	.448	.080
313.50	.431	.080
378.00	.414	.080
404.50	.397	.080
425.50	.379	.080
443.50	.362	.080
448.00	.345	.080
452.00	.328	.080
472.50	.310	.080
514.50	.293	.080
543.00	.276	.080
579.50	.259	.080
644.50	.259	.060
689.50	.241	.060
734.00	.224	.060
787.50	.207	.060
867.50	.190	.060
950.00	.172	.060
980.50	.155	.060
1021.00	.155	.040
1092.50	.138	.040
1303.50	.138	.020
1479.50	.138	.000
1487.50	.121	.000
1494.00	.103	.000
1611.00	.086	.000
2439.50	.069	.000
3173.00	.052	.000
3665.00	.034	.000
5964.00	.017	.000
7792.00	.000	.000

Bold text indicates the cut-off that is used to differentiate active infection from exposure.

**Figure 3:**
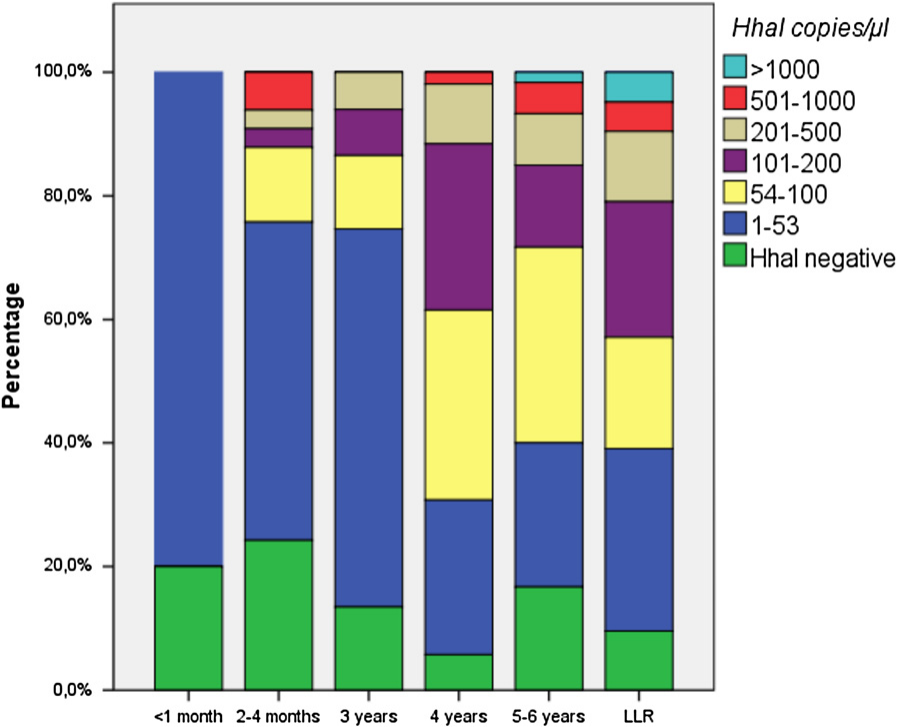
**Stratification of *B*. *malayi HhaI *copies/μl in transmigrants and life-long residents.** Transmigrants resident <3 years had significantly fewer *HhaI* copies/μl than those resident >4 years, who had *HhaI* loads similar to LLR (Cochran-Armitage test for trend *P* < 0.001).

A more detailed analysis was performed by quantifying the *HhaI* gene in the transmigrant samples, sorting them by time of residence and using the cutoff to assign infection status. A positive *HhaI* qPCR result could indicate exposure to larvae (L3) (< 53 *HhaI* copies/μl, below cutoff) or infection with adult worms that may or may not release Mf (>53 *HhaI* copies/μl, above cutoff). In the transmigrants present for less than one month all individuals had <53 *HhaI* copies/μl (Table 4, Figure 3). In the 2–4 months and 3 years group, 24-25% had *HhaI* levels above the cutoff, whereas IgG4 results, which detect host antibodies to adult worms and Mf, were first seen after 3 years residence (7%). After living for 4 years in the endemic area, 69% of the transmigrants had *HhaI* copy numbers above the cutoff, but only 12% were detected by the IgG4 antibody test. After 4 years residence, *HhaI* copy numbers in 62% of the transmigrants were similar to those of LLR. The IgG4 test results in 42% of the transmigrants were similar to those of LLR after 5 years of residence. Dividing the transmigrant population into two groups (residence < 3 and > 3 years) there was a significant trend for higher *HhaI* copy numbers in the group living more than 3 years in the endemic area (Cochran-Armitage test for trend *P* < 0.001). Additionally the number of individuals showing PCR results above the cutoff (53 *HhaI* copies) was significantly increased in the 4–6 year group (P < 0.001 Fisher’s exact test).

**Table 4:** **Detection of *B*. *malayi HhaI *** **in transmigrants and life-long residents in South Sulawesi, Indonesia**

**Length of residence**	**Analyzed individuals**	**Children/Adults**^**a**^	**Mf prevalence**	***HhaI *** **PCR prevalence > 53 copies (%)**	***HhaI *** **PCR prevalence < 53 copies (%)**^**b**^	**IgG4 BmR1 prevalence (%)**
≤ 1 month	15	1/14	- ^c^	0/15 (0)	12/15 (80)	0/15 (0)
2-4 months	33	10/23	- ^c^	8/33 (24)	17/33 (52)	0/33 (0)
3 years	67	34/33	0^d^	17/67 (25)	41/67 (61)	5/67 (7)
4 years	52	19/33	0	36/52 (69)	14/52 (27)	6/52 (12)
5 - 6 years	60	28/32	5	36/60 (60)	14/60 (23)	25/60 (42)
LLR	105	71/34	14	65/105 (62)	30/105 (29)	42/105 (40)
**Total**	**334**	**165/169**	**19**			

^a^Children ≤ 15 years, adults 16 years + .

^b^All samples < 2 *HhaI* copies/μl were considered PCR negative (detection limit), all samples < 53 *HhaI* copies/μl were considered as not infected.

^c^For logistic reasons blood was collected during daytime in 2 villages, therefore Mf counts are absent.

^d^Mf prevalence in the 3-year residents could be determined in 20 individuals only.

In summary these results indicate that in Mf-negative individuals the PCR has a higher rate of detection than IgG4 serology in transmigrants. After 3 or more years of residence in the endemic area, the copy numbers for *HhaI* and the respective rate of active infection increases. In all groups we found samples with low *HhaI* copy numbers (<53 copies). We conclude that, in most cases, these individuals have been exposed to infective larvae but have no active worm infection.

## Discussion

Filariasis experts have requested the development of tests that can detect infection in individuals with ultra-low Mf densities or cryptic infections [29]. In recent years, improved diagnostic methods became available for brugian infections, but were mainly based on Mf positive samples. To meet the request for tests that detect infections when Mf are no longer detected, e.g. due to successful MDA, we modified the *HhaI* QuantiTect® PCR, previously developed by Rao *et al*. [19] by using a novel mastermix setup that allowed us to increase the sample DNA volume to 50% of the reaction volume. We validated the new assay with a *B. malayi* animal model and a unique sample set from an Indonesian transmigrant population with a known history of time spent in an endemic area. In our experiments, the optimized qPCR assay was sensitive at detecting *Brugia* DNA in pre-patent amicrofilaremic animals, Mf-positive patients from Central Sulawesi and in a large set of amicrofilaremic patients from South Sulawesi. These results are novel to previous studies, as we were especially focusing on Mf-negative samples with a pre-patent, latent or cryptic/occult infection. These groups are often seen in the related *W. bancrofti* infections for which a specific antigen test is available.

The 320-bp *HhaI* tandem repeat sequence of *B. malayi* has been used as a target for *B. malayi* PCR assays for many years [19,20]. Rao *et al*. found that detection of the *Brugia HhaI* repeat was as sensitive as microscopic detection of Mf analyzing mainly Mf-positive samples from a highly endemic area [19]. Additionally, the real-time PCR assay showed higher sensitivity compared to conventional PCR and TaqMan PCR assays and detected DNA of nocturnally periodic *B. malayi* in day blood samples. This represented a significant improvement over previously available diagnostic methods in brugian filariasis. The *HhaI* repeat is specific for brugian filariasis and is not detected in *W. bancrofti* infections, which is also endemic in South East Asia. The PCR does not cross-react with other common worm infections, further underscoring the specificity of the assay as seen in our results and by Rao *et al*. [19]. We were able to confirm the sensitivity of the assay in different states of infection using samples from *B. malayi* infected but pre-patent jirds, from uninfected jirds and samples from patients with LE who were mostly amicrofilaremic and possibly without adult worm infection (assuming similar infection kinetics as with bancroftian LE).

The question arose whether the qPCR was able to detect circulating worm DNA in plasma without circulating Mf, a case that would be seen in pre-patent infections and chronic pathology such as LE. From our results we concluded that this was the case, and it is also in concordance with other worm infections, e.g. schistosomiasis in which a positive PCR result was seen while worms were establishing liver infection, i.e. in pre-patency [30].

LF is mainly diagnosed by direct microscopic demonstration of Mf in peripheral night blood of infected people. However, this method does not detect amicrofilaremic individuals with adult worm infection, *i.e*., latent or pre-patent infections, who are still at risk of developing pathology. For *W. bancrofti* infections, assessment of antigenemia using the ICT® or TropBio® ELISA test kits is available to detect infections in amicrofilaremic individuals and offers the convenience of “any-time-of-day testing” [31]. It could be shown in bancroftian filariasis that approximately twice as many individuals are CFA + than are microfilaremic [11]. Ultrasonography (USG), an additional tool for *W. bancrofti* diagnosis, can be used to detect adult filarial worms by their “filarial dance sign” [32]. In brugian filariasis, accounting for 10% of the 120 million worldwide LF infections, no antigen test is available and attempts to reliably detect adult filariae with USG examinations have failed [33] or did not allow reliable, frequent detection [12].

To get a more detailed view of the sensitivity of the *HhaI* assay, particularly in amicrofilaremic infections, we analyzed a large number of individuals that had moved from a non-endemic area to a *B. malayi* endemic area. In the group of individuals who were in the endemic area less than four years, our results showed a very high *HhaI* positive rate (up to 80%), whereas none of these individuals were IgG4 positive. The high rate of detection in this group of transmigrants was unexpected. We concluded that the positive PCR results were due to exposure to infective larvae, not to active infections with adult worms. As the *HhaI* copy numbers were very low in these groups we defined a threshold which could discriminate between exposed and infected individuals based on real-time PCR results.

For monitoring *Brugia* infections using day samples we used ROC-curve analysis to set the threshold associated with active infection to >53 *HhaI* copies/μl in a reaction using 10 μl of plasma DNA and the QuantiTect® Virus NR Kit. This analysis predicted a 74.1% true-positive result for detecting an active infection in Mf-negative people. To our knowledge, this is the first time that results of qPCR in *B. malayi* infections have been used to discriminate between active adult worm infections and exposure to infective L3 larvae. The threshold found in our results may require a trial at other endemic sites, or a multicenter trial, to confirm the current cutoff. Comparing LLR to the transmigrants present in the endemic area for 6 years, the percent of Mf-positives are 13% and 12%, respectively, showing that over time, the infections detected in the transmigrant population are not false alarms. It is also possible to set a higher threshold based on the coordinates of the ROC curve. For example a threshold set at 100 *HhaI* copies for active infections, with a decreased true positive rate (62%) and respectively decreased false positive rate (12%), would reflect more or less the results of the IgG4 antibody test. If the threshold is set at 53 *HhaI* copies, the likelihood to detect exposed but not yet infected individuals is higher. The results show that PCR cannot replace individual diagnostics for the determination of infection status, but could help mapping endemic areas which is important for monitoring mass drug administration (MDA) programs [9,18,19].

The discrepancy of PCR and IgG4 results in the first years of living in an endemic area may be due to the PCR sensitivity for incoming larvae, most of which will not develop into an active infection. Edeson *et al*. showed in animal experiments with *B. malay*i that only 13% of over 400 injected larvae develop into adult worms [34]. The 87% of the larvae that are killed would then be detected by PCR and do not reflect active infection. The parasite burden in the first years after transmigration is probably not high enough to produce detectable IgG4 antibodies, whereas PCR is able to detect parasite DNA in individuals that are exposed to infective larvae. Notably there were individuals with high *HhaI* copies/μl who were IgG4 negative. Based on serology they would have been diagnosed as *Brugia* negative even though it is likely they have pre-patent or at one time had active infections. Conversely, however, there were also 19 IgG4 positive individuals that were diagnosed as PCR-negative, indicating previous exposure to filarial worms or larvae but no current active infection. Regarding the population dynamics of *B. malayi* our qPCR results showed that *HhaI* copies/μl increase with length of time in the area, and indicate the development of active infections.

Introduction of the CFA test for *W. bancrofti* infections showed that 50% of the population in an endemic area had a latent infection. In our study with a highly sensitive and specific qPCR targeting the *B. malayi HhaI* repeat sequence we observed a similar level of latent infections with *B. malayi* infections that, importantly, would not be detected by IgG4 serology or microscopy. Compared to an antigen test used for *W. bancrofti* infection the qPCR is more expensive, but there is currently no other method available for detecting *B. malayi* infections in Mf-negative persons with latent infections.

## Conclusions

The modified real-time PCR for the *HhaI* repeat sequence, which is highly sensitive and specific for *B. malayi*, may become an effective method for monitoring *B. malayi* control programs. Due to possible development of severe LF pathology such as lymphedema, the ability to determine the status of infection, especially in amicrofilaremic patients, as early as possible could lead to prevention of disease progression in individuals of endemic areas. Additionally, identifying possible recrudescence after MDA early could help local health services intervene and prevent wide-spread recrudescence that might otherwise require several years of MDA to be implemented. In the absence of an antigen test for brugian infections the optimized *B. malayi HhaI* PCR can be used to identify infected Mf-negative individuals and should also be useful for assessing the success of MDA in the framework of the Global Programme to Eliminate Lymphatic Filariasis.

## Competing interests

The authors declare that they have no competing interests.

## Authors’ contributions

AA participated in the study design, conducted laboratory tests and statistics, interpreted laboratory results, wrote and edited the manuscript. ES participated in organization and design of the study and provided patient samples. SW participated in organization and design of the study and provided patient samples. MY participated in organization and design of the study and provided patient samples. RMM conducted animal experiments and provided animal samples. UKS participated in statistical analysis of the data. KP participated in the study design, conducted laboratory tests, edited and submitted the manuscript. AH conceived the study, and wrote and edited the manuscript. All authors read and approved the final version of the manuscript.

## Supplementary Material

Additional file 1 **Flow Chart comparing the sensitivity of *HhaI *** **PCR and IgG4 BmR1 dipstick test.** Click here for file

Additional file 2 DNA extraction and real-time PCRs. Click here for file

Additional file 3 **Detection limit of the *B. malayi HhaI *** **real-time PCR in the 2 μl and 10 μl assays.** Click here for file

Additional file 4 **Validation of the *HhaI *** **PCR with samples from *B. malayi *** **LE and Mf positive volunteer DNA samples.** Click here for file
