# Supplementary figures and images for: Real-time PCR detection of the HhaI tandem DNA repeat in pre- and post-patent Brugia malayi infections: a study in Indonesian transmigrants
Source: Parasit Vectors. 2014 Mar 31;7:146. doi: 10.1186/1756-3305-7-146 (PMC4021971; doi:10.1186/1756-3305-7-146)

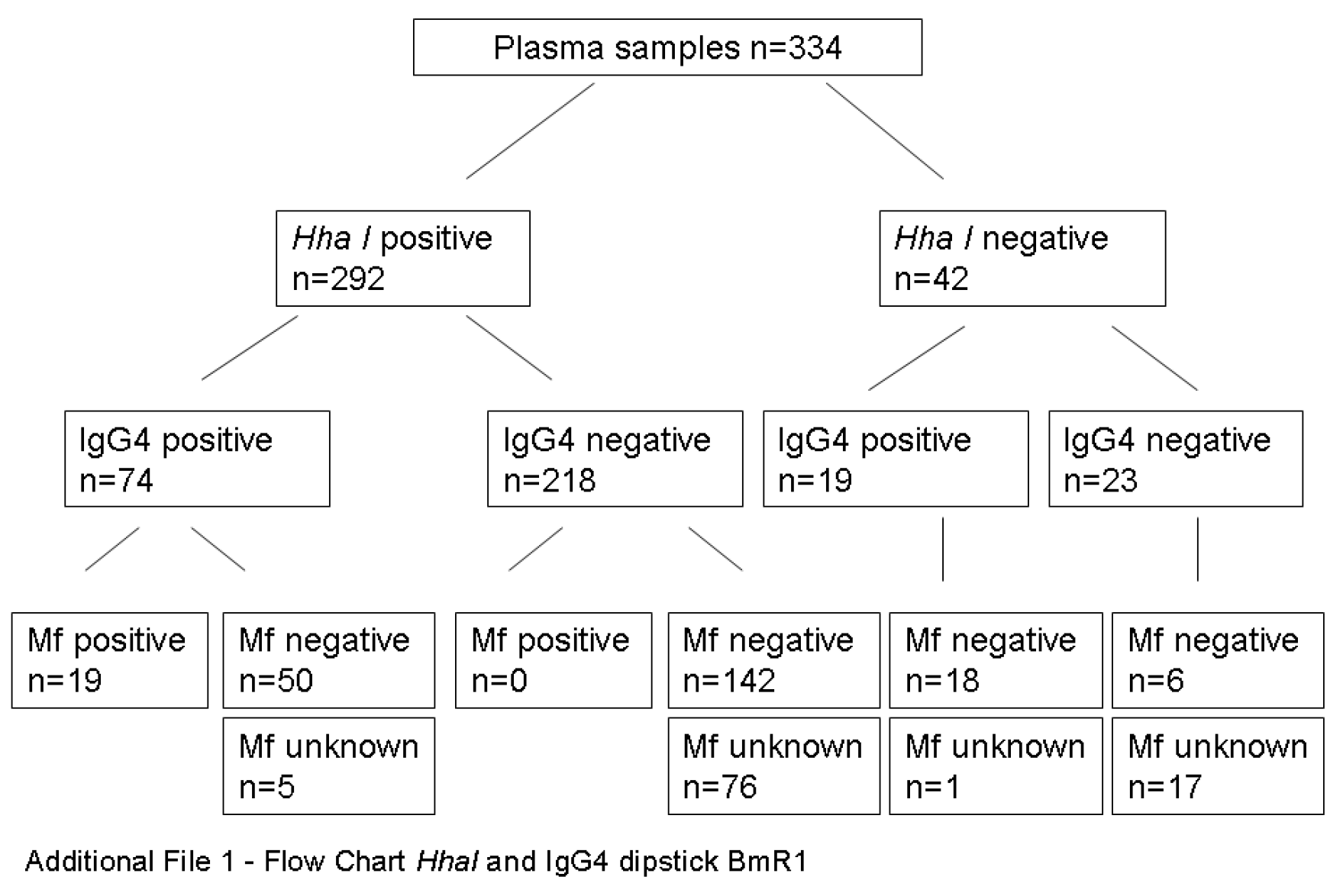


## Additional File 1 – Flow Chart comparing the sensitivity of *HhaI* PCR and IgG4 BmR1 dipstick test

Supplement: Additional file 1 — Flow Chart comparing the sensitivity of HhaI PCR and IgG4 BmR1 dipstick test. [file 1756-3305-7-146-S1.doc]
