# Supplementary material for: Real-time PCR detection of the HhaI tandem DNA repeat in pre- and post-patent Brugia malayi infections: a study in Indonesian transmigrants
Source: Parasit Vectors. 2014 Mar 31;7:146. doi: 10.1186/1756-3305-7-146 (PMC4021971; doi:10.1186/1756-3305-7-146)
Supplement: Additional file 2 — DNA extraction and real-time PCRs. [file 1756-3305-7-146-S2.doc]

**Additional File 2.** DNA extraction and real-time PCRs

Primer and hybridization probe sequences were as published in Rao *et al*. [18]. The *HhaI* repeat (M12691 and AF499109 to AF499129) is arranged as a tandem repeat and is present in several thousand copies per haploid genome. Furthermore, most conventional PCR assays for *Brugia* spp. have used this target, and no consistent sequence variation has been reported in various strains of *B. malayi* and *B. timori*.

For generating the external standard curve, the highest dilutions were measured with five replicates, while the lowest dilutions (2 copies/µl) were measured with 10 replicates in three separate runs. All other PCRs were carried out in duplicate.

The reaction mixture for the real-time PCR consisted of 10 µl of 2x QuantiTect® Probe PCR Master Mix (containing HotStar® Taq DNA Polymerase, QuantiTect® Probe PCR buffer, dNTP mix and 8 mM MgCl_2_), 1 µl 20x Primer Mix (5’-GCAATATACGACCAGCAC-3’ forward and 5’-ACA*TTAGA*CAAGGAAATTGGTT-3’ reverse primer (*denotes incorporation of a supernucleotide) and 0.5 ml 20x QuantiProbe® solution (3’FAM-TTTTTAGTAGTTTTGGC-Eclipse^TM^ Dark Quencher 5’) in a 20 µl reaction. Cycling conditions on a RotorGene 6000 (Corbett Research, Sydney, Australia) included an initial activation for 15 min with 95 °C and 45 cycles of 15 sec at 94 °C, 30 sec at 56 °C and extension for 30 sec at 76 °C. Fluorescence was acquired on the FAM (green) channel during the annealing step at 56 °C.

For the samples with a very low DNA content and which were negative in a first PCR were measured again using a second PCR assay with increased volume of DNA template, QuantiTect® Virus NR. The reaction mixture contained 4 µl of 5x QuantiTect® Virus NR mastermix (containing HotStar® Taq Plus DNA Polymerase, QuantiTect® Virus PCR buffer, dNTP mix and MgCl_2_), 1 µl Primer Mix 20x and 0.5 ml 20x QuantiProbe® solution and 10 µl DNA sample in a 20 µl reaction. The profile on a RotorGene 6000 included an initial activation for 5 min at 95 °C and 45 cycles of 15 sec at 94 °C and 30 sec at 60 °C. Fluorescence was acquired on the FAM channel during the annealing step at 60 °C.

To test for any inhibition factors in the extracted plasma samples, a third PCR using a murine *IFN-γ* plasmid was added to each DNA sample. For the reaction, a master mix containing 1X HotStar® Taq Polymerase buffer (Qiagen), 200 µm dNTP, 400 nM each of forward (3’- TCAAGTGGCATAGATGTGGAAGAA-5’) and reverse (3’- TGGCTCTGCAGGATTTTCATG-5’) primer, 0.2 µl SYBR® Green (1:1000 diluted in DMSO, Roche, Manheim, Germany), 2.5 units HotStar® Taq, 2 µl reference plasmid DNA and 2 µl sample DNA in a 20 µl reaction. The PCR profile was: 1x15 min at 95°C, 45 cycles of 94 °C for 15 sec, 58 °C for 20 sec, 72 °C for 20 sec. Fluorescence was acquired on the FAM channel. Samples were considered uninhibited when the reactions containing patient DNA were detected at the same level as the reference plasmid control sample.

Finally, as an internal control of DNA content, the human *β-actin* gene was measured. The master mix contained 1X HotStar® Taq Polymerase buffer (Qiagen), 200 µm dNTP, 300 nM each of forward (3’-GATGAGATTGGCATGGCTTTA-5’) and reverse (3’-AACCGACTGCTGTCACCTTC-5’) primer, 0.2 µl SYBR® Green (1:1000 diluted in DMSO, Roche, Mannheim, Germany), 2.5 units HotStar® Taq Polymerase, and 2 µl DNA in a 20 µl reaction. The gene was amplified in a RotorGene 6000 (Corbett Research) using the following conditions: 1x15 min at 95 °C, 35 cycles of 94 °C for 15 sec, 58 °C for 15 sec and 72 °C for 15 sec. Fluorescence was acquired on the FAM channel at 58 °C. To exclude unspecific SYBR® Green products, a melting curve analysis from 62-99 °C was performed after each run. Only when a sample was positive for *β-actin* and shown to have no inhibitors, was an *HhaI* reaction with no signal considered as negative.
