# Supplementary material for: Real-time PCR detection of the HhaI tandem DNA repeat in pre- and post-patent Brugia malayi infections: a study in Indonesian transmigrants
Source: Parasit Vectors. 2014 Mar 31;7:146. doi: 10.1186/1756-3305-7-146 (PMC4021971; doi:10.1186/1756-3305-7-146)
Supplement: Additional file 3 — Detection limit of the B. malayi HhaI real-time PCR in the 2 μl and 10 μl assays. [file 1756-3305-7-146-S3.doc]

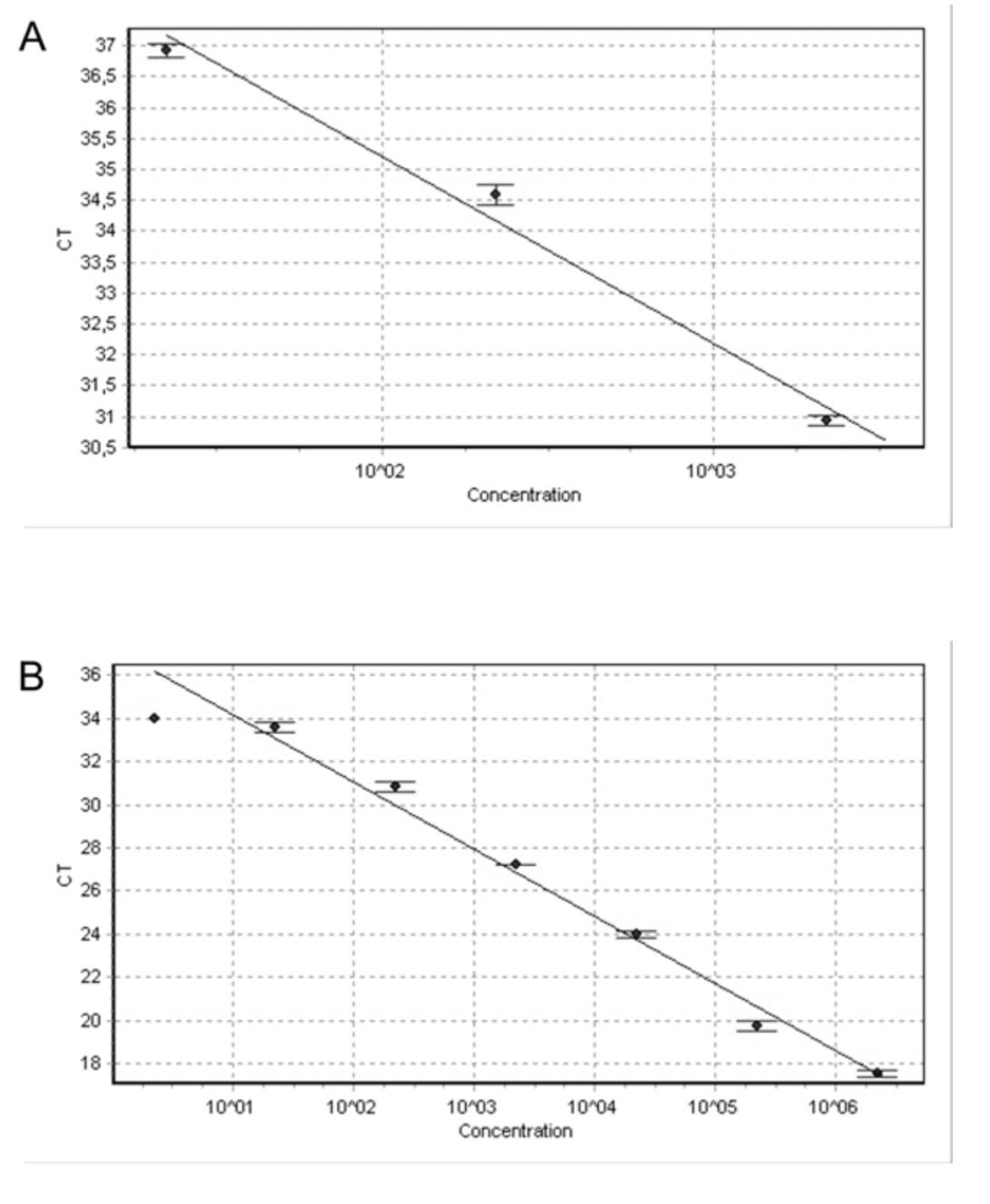


## Additional File 3 – Detection limit of the *B. malayi* *HhaI* real-time PCR in the 2 µl and 10 µl assays. A) Sensitivity of the *HhaI* PCR assay using 2 µl of DNA as template. The detection limit of the *HhaI* PCR was determined with a plasmid dilution series using 2-2000 copies/µl. Each sample contained 2 µl of diluted plasmid in a final reaction volume of 20 µl using the QuantiTect® Probe PCR Master Mix, Qiagen. In the lowest dilution (2 copies/µl, N=10), 50% were detected. In the second lowest (20 copies/µl, N=5) 80% of the samples had a positive signal. With the 200 copies/µl dilution (N=10), 100% of the plasmid samples had a positive signal. The PCR reaction efficacy was 85%. The standard curve with standard error of mean is shown. B) Sensitivity of the *HhaI* PCR assay using 10 µl of DNA as template. Each PCR replicate used 10 µl of diluted plasmid as the DNA target in a final 20 µl reaction volume QuantiTect® Virus NR Master Mix as described in Additiona File 2. 100% of the the replicates of all dilutions were detected when using the higher DNA volume in the reaction. The standard curve with standard error of mean is shown.
