# Supplementary material for: Real-time PCR detection of the HhaI tandem DNA repeat in pre- and post-patent Brugia malayi infections: a study in Indonesian transmigrants
Source: Parasit Vectors. 2014 Mar 31;7:146. doi: 10.1186/1756-3305-7-146 (PMC4021971; doi:10.1186/1756-3305-7-146)
Supplement: Additional file 4 — Validation of the HhaI PCR with samples from B. malayi LE and Mf positive volunteer DNA samples. [file 1756-3305-7-146-S4.doc]

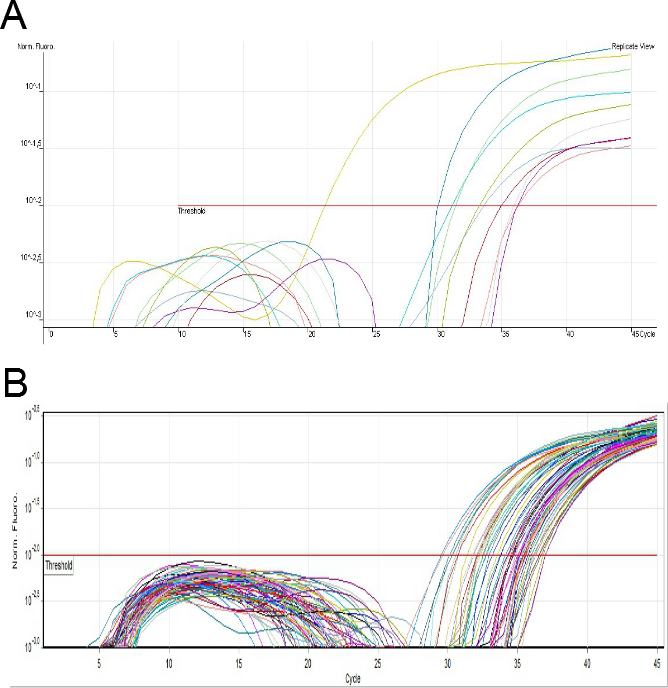


## Additional File 4 – Validation of the *HhaI* PCR with samples from *B. malayi* LE and Mf positive volunteer DNA samples. A) Ct values of nine elephantiasis patients. One of the patients was Mf positive (1 Mf/ml), eight were Mf negative. B) Ct values of 30 Mf positive samples (Median Mf/ml 305, range 9-1436). Each 20 µl PCR reaction used 10 µl of DNA extracted from the respective plasma samples with the QuantiTect® Virus NR Master Mix. The reaction was performed as in Additional File 2.
